# Transcriptomic changes in the jerboa supraoptic nucleus during osmotic stress: PCSK1 and PTPRN changes are conserved across desert and mesic species

**DOI:** 10.1111/jne.70233

**Published:** 2026-07-26

**Authors:** Benjamin T. Gillard, Nabil Amor, Michael P. Greenwood, Abdulaziz N. Alagaili, David Murphy

**Affiliations:** ^1^ Molecular Neuroendocrinology Research Group, Bristol Medical School, Translational Health Sciences, Dorothy Hodgkin Building University of Bristol Bristol UK; ^2^ LR18ES05, Laboratory of Biodiversity, Parasitology and Ecology of Aquatic Ecosystems, Department of Biology—Faculty of Sciences of Tunis University of Tunis El Manar Tunis Tunisia; ^3^ School of Psychology and Neuroscience University of Bristol Bristol UK; ^4^ Department of Zoology King Saud University Riyadh Kingdom of Saudi Arabia

**Keywords:** cross species comparison, *Jaculus jaculus*, osmoregulation, supraoptic nucleus, WGCNA

## Abstract

The supraoptic nucleus (SON) is a hypothalamic region essential for the orchestration of osmoregulation in mammals. We sought to understand how gene expression in the SON is orchestrated in a rodent species adapted for survival in arid desert conditions. After an acclimatisation period, we compared the male and female trancriptomes of the Lesser Egyptian jerboa (*Jaculus jaculus*) SON under euhydrated, dehydrated, and rehydrated conditions. Gene expression in the jerboa SON was remarkably stable across the conditions with only 46 differentially expressed genes (DEGs) after dehydration and 4 DEGs after rehydration compared to controls; far fewer than previously shown to respond in the jerboa kidney and in the SON of mesic species. When comparing across species (jerboa, camel, and rat), the genes PCSK1 and PTPRN were consistently regulated under conditions of osmotic stress suggesting they have important conserved functions. The dehydration response in jerboa SON is tightly regulated. PCSK1 and PTPRN are identified as conserved components of the response, indicating their importance.

## 1 INTRODUCTION

The magnocellular neurons (MCNs) of hypothalamic SON are primarily tasked with the synthesis and secretion of the neuropeptide hormones arginine‐vasopressin (AVP) and oxytocin (OXT). Synthesised AVP and OXT are transported along MCN axons to storage in the posterior pituitary. A rise in extracellular osmolality, for example evoked by inadequate water intake, provokes release of these hormones into the circulation. At the level of the kidney, AVP encourages water retention, whilst OXT increases sodium excretion (natriuresis) to alleviate hypertonicity.^1^

As a consequence of osmotic stress, the SON undergoes considerable plasticity.^2^, ^3^ We have sought to understand this at the transcriptomic level, and have accumulated considerable data in mesic species.^4^, ^5^, ^6^, ^7^

Diversifying information sources is important to build stronger models of the transcriptomic response in the SON, in particular species that can withstand much longer periods of dehydration than mesic laboratory models. To this end, we recently reported the transcriptome of the dromedary camel SON^8^ under euhydrated and dehydrated conditions. However, there is currently no transcriptomic dataset for the SON in desert rodents which could act as a useful evolutionary bridge between the ungulate camel and the traditional laboratory rodents. We have thus sequenced the SON transcriptome in the Lesser Egyptian jerboa (*Jaculus jaculus*, hereafter referred to as jerboa). The jerboa is a tractable model for water restriction with a published genome^9^ and previous studies demonstrating the physiology behind its ability to survive long stints without water.^10^, ^11^, ^12^ Previously, we have shown water restriction elicits canonical physiological responses such as weight loss and increased blood osmolality in the jerboa along with a substantial transcriptional response in the kidney.^13^

Here, we describe the male and female transcriptomes of the jerboa SON in control euhydrated animals and in animals subject to dehydration followed by rehydration to better understand the central mechanisms regulating water balance in this xeric mammal. We place this new data in the context of previously published SON transcriptomes of the camel and rat to understand conserved mammalian responses.

## 2 METHODS

**FIGURE 1:**
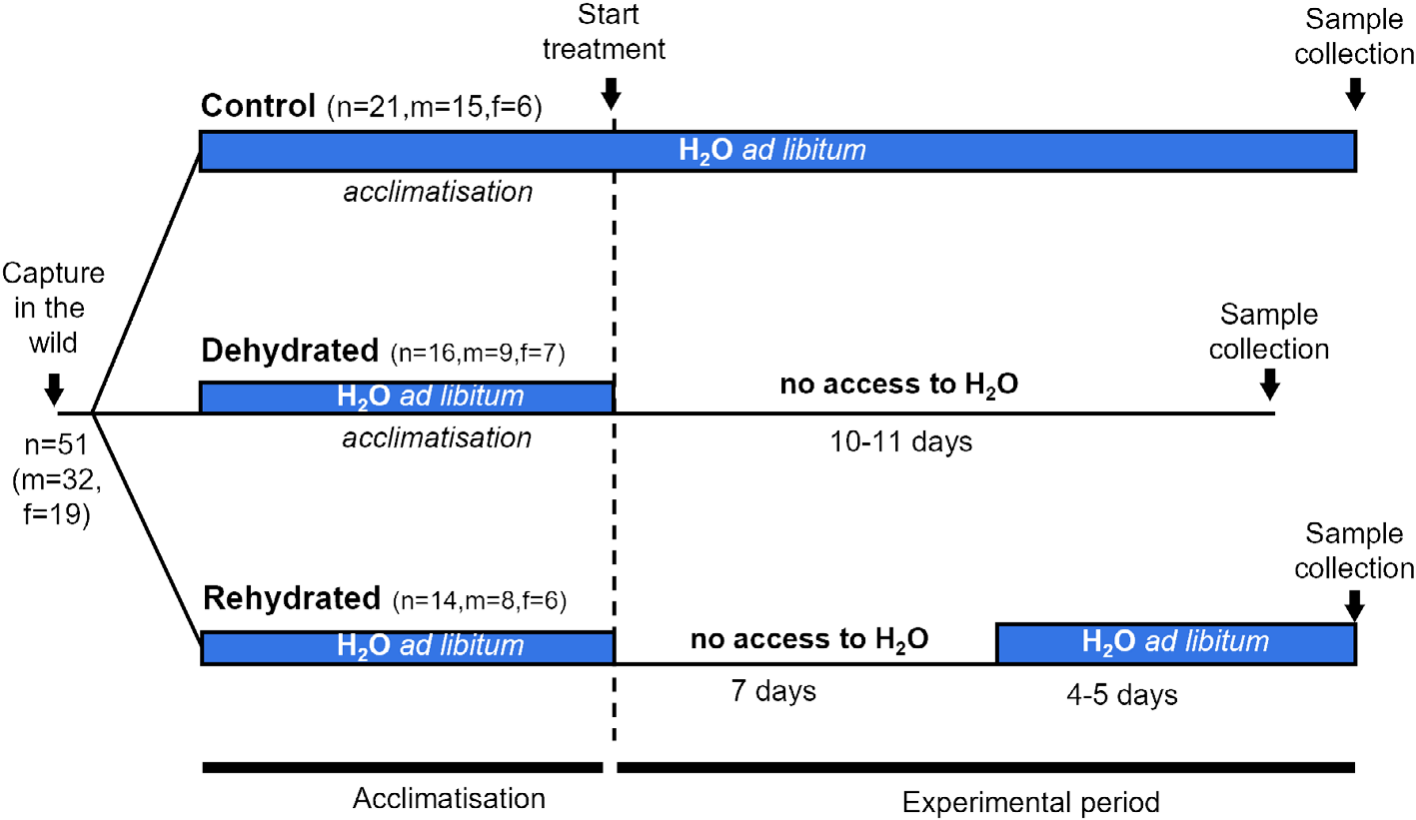
A experimental design. After an acclimatisation period, wild jerboas were put into three experimental groups: Control (water access ad libitum), dehydration (water restriction for 10/11 days), or rehydration (water restriction for 7 days then water access ad libitum for 3/4 days). Samples and measurements were taken from the animals at the end of each group's experimental period.

The general experimental design and animal husbandry are described in detail in a previous study^13^ (see Figure 1 for overview). The length of water restriction was chosen based on animal health and matches other desert rodent studies found in a thorough review of previous water restriction studies^14^ providing insightful data on the osmoregulatory systems of this xeric species compared to mesic species.

### 2.1 Brain mapping by tissue staining

A brain from a dehydrated female jerboa was cut into 20 μm slices using a cryostat (CM3050S, Leica) with a chamber temperature of −20°C. Each slice was thawed onto a microscope slide (Superfrost J1800AMNZ, ThermoFisher).

For immunohistochemistry of AVP, slices were fixed in ice cold 4% w/v filtered Paraformaldehyde (PFA) (28794.295, VWR) for 15 min. Three 5 min phosphate buffered saline (PBS) washes were followed by a blocking step using 3% bovine serum albumin (BSA) (A7906, Sigma) in PBS‐t (0.3% v/v Triton X‐100 (T9284, Sigma); P3813, Sigma) for 20 min. An antibody specific to the neurophysin II segment of the AVP prohormone (MABN845, Sigma, RRID:AB_2819363) was diluted 1:100 in PBS‐t and incubated with the slices for 48 h at 4°C. Three 5 min PBS washes were followed by secondary antibody (Alexa Fluor goat against mouse IgG 488, Santa Cruz) prepared in PBS‐t for 1 h at room temperature (RT) in the dark. Three 5 min PBS washes were followed by 1 min incubation with DAPI (D1306, Invitrogen), then slides were air dried and mounted using Prolong Gold antifade reagent (P36930, ThermoFisher).

Imaging of the slides was performed using a widefield microscope (DMI6000, Leica) at the University of Bristol Wolfson Bioimaging Facility with a monochrome camera (DFC365F, Leica) for fluorescent imaging. Leica LAS‐X acquisition software was used to stitch together images taken from multiple pictures.

### 2.2 RNA extraction from SON

Fresh frozen brains were sliced on the coronal plane at 100 *μ*m on a cryostat. Twelve bilateral punches of the SON were taken using a 1 mm sample core (18035‐01, Fine Science Tools). Punched tissue samples were lysed by adding Qiazol (79,306, Qiagen), then vortexed for 2 min, then incubated on ice for 10 min. Lysed samples were centrifuged at 12,000 × g for 10 min at 4°C and the supernatant was transferred to a new tube. One‐fifth volume of chloroform (99.9%, 22,711.244, VWR) was added to each sample and vortexed for 15 s. Samples were then centrifuged at 12000 × g for 15 min at 4°C. RNA was extracted from the top RNA‐containing aqueous phase using Zymo Direct‐Zol RNA miniprep (R2052, Zymo) as per manufacturer's instructions.

### 2.3 Library preparation and sequencing

The mean RNA concentration from extracted SON samples was 24.99 ng/μL (12.2–38.8) as measured by Qubit High Sensitivity Assay (Q32852, Qubit 4 Fluorometer, ThermoFisher).

Sequencing of the jerboa SON samples was performed at the University of Bristol Genomics Facility. The average library size of each of the 37 male and female SON samples was determined using the Agilent DNA1000 assay and the TapeStation 4150 instrument, before normalising to 4 nM and pooling equimolarly for sequencing.

RNA integrity number (RIN) values ranged from 7.9 to 9.6 for the SON samples with a mean of 9.1. Samples with the highest RIN values were selected for sequencing.

A sample of 100 ng of input total RNA was used in the Illumina Stranded mRNA library preparation with ligation kit (20040534) and followed with a 4 min fragmentation time. IDT for Illumina RNA UD Indexes (20,040,553, Set A) were used during library preparation. Sequencing of 2 × 75 base pair (bp) reads was undertaken using NextSeq500 High Output Version 2.5.

Raw fastq files generated can be found at the Gene Expression Omnibus under the accession GSE242975.

### 2.4 RNAseq analysis

Sequenced reads were aligned/mapped to the latest jerboa genome (JacJac.mCur) using the Rsubread package.^15^ The mean total number of reads for jerboa SON samples was 50,472,036 (88.80%).

FeatureCounts^16^ was used to assign mapped reads to features on the genome. The mean number of assigned fragments in the jerboa SON samples was 15,759,007 (62.37%). These alignment ratios are similar to previous sequencing experiments.

There were no significant differences between the RIN values between the condition (analysis of variance (ANOVA) *p* = 0.16, *F* = 1.983, df = 2) or sex (Welch *p* = 0.07, *t* = −1.8694, df = 22) groups.

Differential expression between the three experimental conditions was estimated using the DESeq2 package.^17^ Sex was correlated with principal component (PC) 7 (0.43) which accounted for 4.4% of variation so there appears to be some influence, albeit smaller, than that driven by the experimental conditions. Ten genes (*p*_adj_ < 0.05) were explained better by the additive model containing sex compared to the simple model with only the condition variable. However, there were 773 genes with an unadjusted *p*‐value <0.05 including OXT. We decided to use an additive model to control for any sex differences because the question posed here is how the experimental conditions affect expression in the jerboa SON. For reference, the additive model comparison between sexes is included in Supporting Information.

### 2.5 Network building with weighted correlation network analysis (WGCNA)

Before building a network with WGCNA, genes that have consistent expression across all samples were removed. Starting with a full dataset of 17,583 genes (which have already been filtered to exclude low expression genes in the DESeq2 analysis), removing missing value genes reduced the number of genes by 10 to 17,573. Removing the bottom 30% of low variance genes reduced the dataset further by 5265 genes to a dataset of 12,308 genes to take into WGCNA. Exclusion of these stably expressed genes reduces noise in the data and does not affect the overall drivers of differences between samples.

### 2.6 Quantitative polymerase chain reaction (qPCR) validation

cDNA was synthesised as per manufacturer's instructions using GoScript (A2801, Promega) with random primers. Primer sequences for jerboa SON genes are shown in Table 1. The most stable reference genes (by RefFinder^18^) are PPIA and CUL1 (comprehensive ranking values 1.57 and 1.68 respectively). Primers were diluted to a final concentration of 300 nM as part of the assay mixture.

**TABLE 1:** Jerboa primer sequences: SON.

Gene	Ensembl	Forward primer	Reverse primer
AVP	ENSJJAG00000024270	TGTCGGACTTGGAGCTGAGA	CAGGTAGTTCTCCTCCTGGCA
CAPRIN2	ENSJJAG00000018136	TGGGAATTACAGCCAGCTACA	GACTCTCTCCACCCTGCTCTT
CUL1	ENSJJAG00000003559	TTGATTCAGGCAGCCATCGT	TAATCACTGGGACTCGGGGT
GLP1R	ENSJJAG00000009597	GGCAGCAGTGTGCAAATGG	GGCGTTTTCTCCTCTCCTCC
hnAVP	ENSJJAG00000024270	TGAGGGCAAAACTAGAGCCG	GCACCACTCTCAGCAGTCTT
NOS1	ENSJJAG00000021583	CATGAAGCCCTCGTTCTGGT	CGACCCTTGTAGCTCTTCCTTT
OXT	ENSJJAG00000023461	GACCTCAGCCTGCTACATCC	ACTTGCGTGCATCGAGGTC
PCSK1	ENSJJAG00000014871	CCCTGGAAGCAAATCCCAATC	CTGGCCAAGGGATCGTACTC
PDYN	ENSJJAG00000024828	TGTCACTCTCTCATTGGCTCC	GCTGTGAGCGCTACCAAATC
PPIA	ENSJJAG00000015423	CATCTGCACTGCCAAGACTG	TTCCTAGACCCAAGGCGTTC
PTPRN	ENSJJAG00000018101	GTGCCCACGGCTGTCTG	AGCGTTGGAGAATTGGGGAG
VIP	ENSJJAG00000018682	CCGCGTACCAATCAAACGAC	CCCATGGGAAACAACAACTCC

*Note*: A soft threshold power of 11 based on the lowest power before reaching a scale free topology value of 0.9. Similar modules (>0.7 correlation) were merged for a total of 19 modules.

Assay was run using SYBR green master mix (A25777, ThermoFisher) and measured on StepOnePlus system (4,376,600, ThermoFisher).

Figure S1 shows the RNA Sequencing (RNAseq) results are successfully validated by qPCR with a significant correlation between the methods in both dehydration (Panel A, *R* = 0.87, *p* = 6.8e‐05, 95% CI [0.7, 0.99]) and rehydration (Panel B, *R* = 0.75, *p* = 0.0021, 95% CI [0.11, 0.96]) compared to control.

### 2.7 Trait data

Trait data including sex, caught weight (used as a control), dissection weight (after experimental conditions), plasma sodium, total protein in blood (used as a control), and calculated plasma osmolality were collected to correlate with the SON transcriptome. Further detail on the methods of collection and descriptive statistics can be found in a previous study.^13^

### 2.8 Posterior pituitary (PP) peptide content

To each dissected PP gland, 500 *μ*L 100 mM HCl (H/1200/PB17, Fisher Scientific) was added. Sonication (2 mm amplification, Soniprep 150, MSEFull power, medium tune) at 4°C was performed for 15 s. This was repeated another 2 times, allowing the sample to remain cold (returned to ice) between each sonication period. Samples were incubated at 85°C in a waterbath for 10 min. Samples were centrifuged at 3000 × g at 4°C for 60 min and the supernatant was collected. Enzyme linked immunoSorbent assays (ELISAs) for AVP (#ADI‐900‐017A, Enzo Life Sciences) were performed as per the manufacturer's instructions. Samples were diluted 1:3000 in assay buffer prior to assay. The *R* ^2^ value for assay standard curves was 0.99. Average coefficient of variance for absorbance between sample duplicates was 7%. Data for AVP content in the PP is shown in Figure S2.

### 2.9 Cross species analysis

Table S1 summarises details of samples, sequencing, and data processing steps for each of the three species used in the comparison.

Data for DEG comparison was in the form of DESeq2 output from published data. Differential expression data for camel and rat SON was extracted from supplementary data S2 in Lin et al.^8^ For WGCNA consensus networks, raw read data from featureCounts was extracted from GSE175461 for rat SON. Data for the camel SON was provided by Panjiao Lin from her thesis,^19^ which included a rehydration group. All read data was normalised using a variance stabilising transformation (using a function from DESeq2 package^17^). Consensus network construction created a signed network using bicor correlation. Similar modules were merged with >0.7 similarity and the minimum module size was 30 genes. Networks for each species were calibrated by the single quantile (0.95), this adjusts matrices to make them more compatible.

Venn diagrams were generated using the venn.diagram package^20^ in R.

### 2.10 Statistical tests

All statistical tests were carried out in R using the stats package. One‐way ANOVA was used to show differences between the three groups for RIN and AVP PP measurements.

Spearman correlation was used to show correlation between qPCR and RNAseq data.

## 3 RESULTS

### 3.1 Mapping the jerboa SON

**FIGURE 2:**
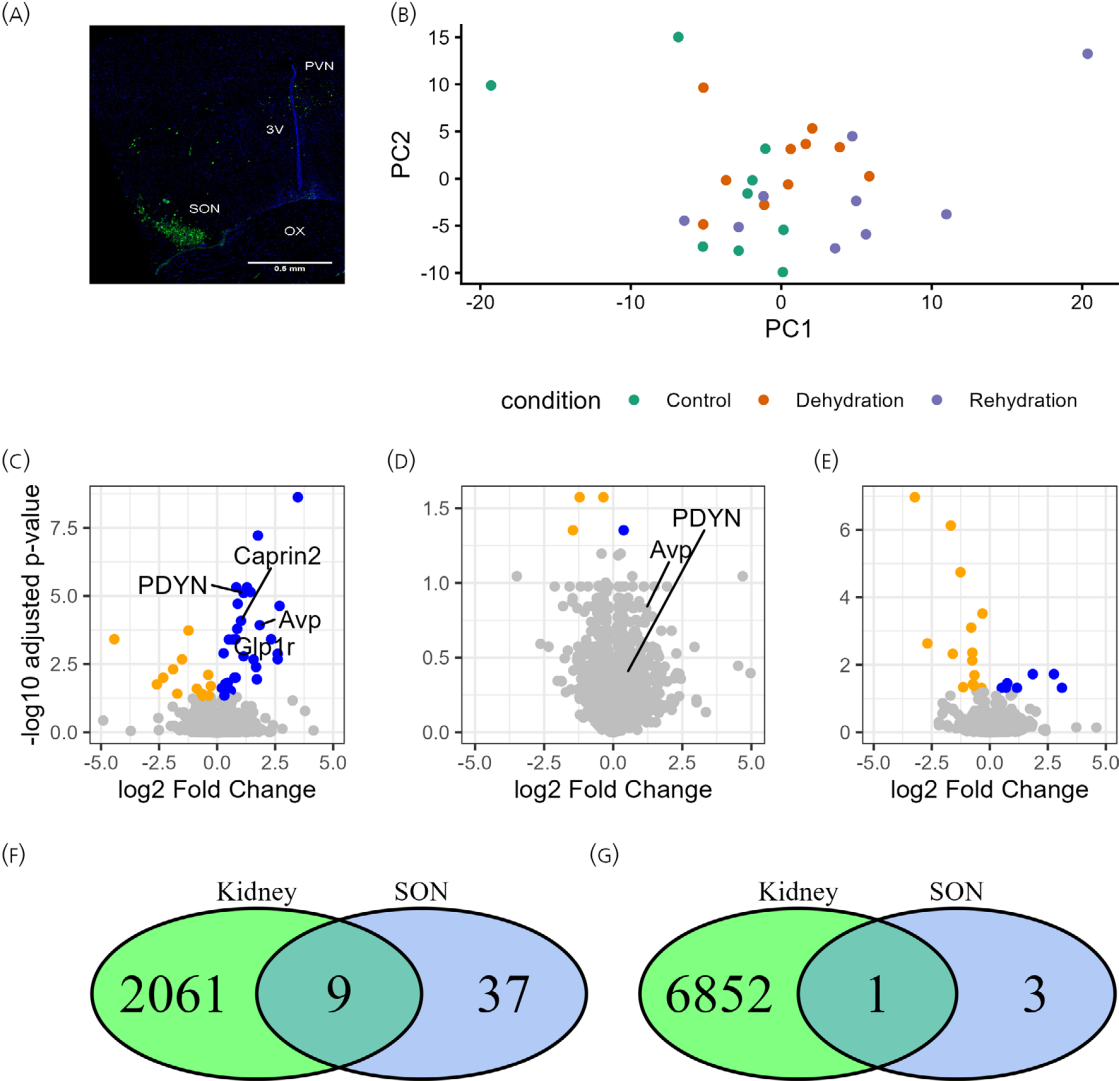
(A) Mapping of the jerboa SON using AVP targeted immunofluorescence. (B) PCA plot of sequenced jerboa SON samples. (C) Volcano plot of genes in the dehydration versus control comparison. (D) Volcano plot of genes in the rehydration versus control comparison. (E) Volcano plot of genes in the rehydration versus dehydration comparison. Genes with *p*_adj_‐value <0.05 are highlighted; orange if downregulated and blue if upregulated. (F) Number of DEGs (<0.05) in jerboa SON and kidney following dehydration. (G) Number of DEGs (<0.05) in jerboa SON and kidney following rehydration.

AVP staining was used to identify the SON (Figure 2A). The SON can be seen as a dense population of cells sitting atop the optic chiasm in the jerboa brain. This is a similar position to that seen in the mouse and rat. In addition to SON, there is some AVP expression in collateral projections and the paraventricular nucleus (PVN) alongside the top of the third ventricle (Figure 2A). This mapping enabled accurate punching of SON tissue.

### 3.2 SON transcriptome in the jerboa

The experimental conditions did not cluster samples clearly (Figure 2B) on a principal component analysis (PCA) plot. PC1 was somewhat correlated with the experimental groups (condition), but this was not a significant association.

In response to dehydration, 46 DEGs were found in the jerboa SON, of which 33 (71%) were upregulated and 13 (29%) were downregulated compared to control (Figure 2C). When water was made available again, there were four DEGs compared to the control group (Figure 2D) with a single gene upregulated and three genes downregulated. There were 21 DEGs when comparing rehydration to dehydration condition, of which 7 (33.33%) were upregulated and 14 (66.66%) were downregulated. The only gene to be differentially regulated (downregulated) in both dehydration and rehydration was KANK1 (ENSJJAG00000018774) which encodes a protein implicated in actin depolymerisation and DNA repair pathways.^21^

No Gene Ontology (GO) terms were significantly enriched (*p* < 0.05) when using DEG lists for over representation analysis (ORA) against a background of all detectable genes in the jerboa SON. When the query list is ordered by *p*_adj_ value (akin to gene set enrichment analysis [GSEA]), DEGs from the control versus dehydration comparison were enriched for a single GO term—extracellular region (GO:0005576, *p* = 3.507e‐04). This term was also enriched in the DEG list from the dehydration versus rehydration comparison (*p* = 1.778e‐02).

The jerboa SON had much fewer DEGs than the jerboa kidney^13^ during dehydration and rehydration (Figure 2E). When comparing DEGs from the jerboa whole kidney (male samples only) and the jerboa SON (male and female samples, additive model), there were nine genes that were significantly regulated by the dehydration condition and one gene (KANK1/ENSJJAG00000018774) that was significantly regulated by the rehydration condition compared to control (Figure 2F).

### 3.3 Differentially expressed messenger RNAs (mRNAs)

AVP is central to osmoregulation in mammals and was differentially expressed in the jerboa SON (as well as heteronuclear RNA, Figure S1). AVP, along with others, was validated by qPCR (Figure S1).

Other genes that are consistently activated in the rat SON by osmotic stress were shown to be significantly changed in dehydration in the jerboa SON. PDYN (ENSJJAG00000024828^22^), CAPRIN2 (ENSJJAG00000018136^23^), GLP1R (ENSJJAG00000009597^24^), and VGF (ENSJJAG00000027841^25^) all followed the same expression pattern in the jerboa SON as that seen in rat data. There were also surprising differences whereby non‐significant changes in expression were observed for genes responsive in the rat SON including CREB3L1 (ENSJJAG00000014152^26^), its upstream regulator NR4A1 (ENSJJAG00000016904^27^), OPN3 (ENSJJAG00000020588^28^), and Rasd1 (ENSJJAG00000021532^29^).

### 3.4 Network analysis of the supraoptic nucleus transcriptome

**FIGURE 3:**
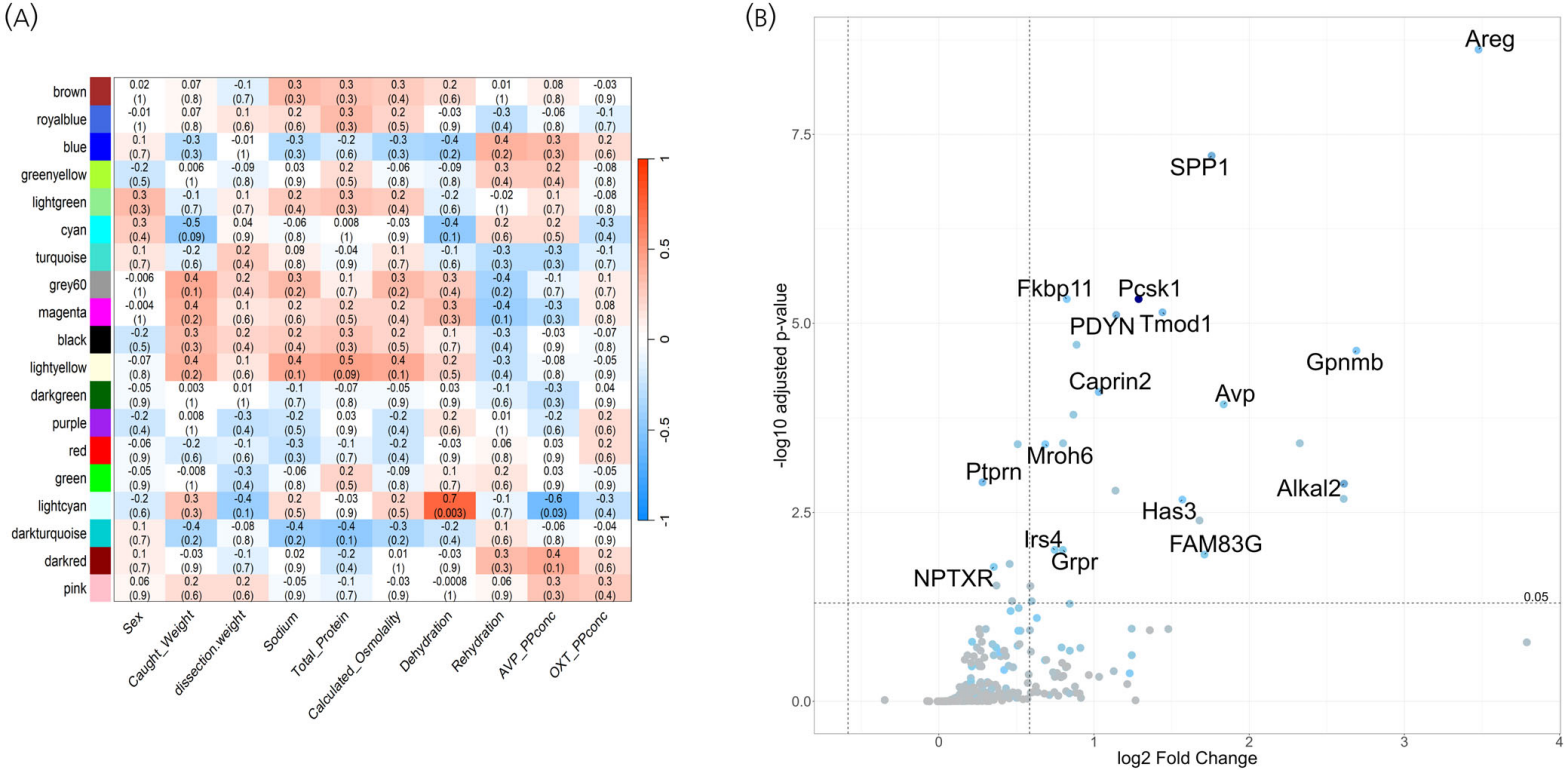
(A) Correlation between jerboa SON module eigengenes and external traits of interest. Correlation is calculated using biweight midcorrelation. The *p*‐values are shown in brackets underneath the correlation value. Red indicates a positive correlation. Blue indicates a negative correlation. (B) Volcano plot of dehydration versus control comparison with genes showing high connectivity within the ‘lightcyan’ module. AVP had high intramodular connectivity (kwithin) is represented by the colour of the data point. PP, posterior pituitary.

WGCNA is used to find modules of closely related genes that can be correlated with physiological traits. Each module has an abstract representation of the expression of genes within a module called an eigengene. Figure 3A shows the correlation of each module eigengene to a selection of trait data where red indicates a positive relationship and blue represents a negative relationship.

The distribution of connectivity for genes within each module suggests the presence of a small proportion of highly connected hub genes. Genes with high connectivity are often strongly correlated with traits linked to the module eigengene.

The ‘light cyan’ module contained 276 genes and is characterised by an increase in expression during water restriction returning to control levels after rehydration. 10.9% of genes in the ‘light cyan’ module were DEGs following dehydration, all of which were upregulated, and there were no DEGs in the ‘light cyan’ module from the rehydration versus control comparison. The trait‐expression heatmap in Figure 3A shows significant correlation between the ‘light cyan’ module eigengene and dehydration. If genes from the ‘light cyan’ module are isolated, PCA shows a significant negative correlation (Spearman, −0.48, *p* < 0.01) between PC1 and dissection weight (weight loss is seen with dehydration). AVP belongs to the ‘light cyan’ module. There is significant negative correlation between the ‘light cyan’ eigengene and PP AVP content (Spearman, −0.6, *p* = 0.03, see Figure S2 for AVP content), suggesting replenishment of AVP stock released from the PP. There were no significantly enriched GO terms by ORA for genes in the ‘light cyan’ module. As AVP is central to the osmoregulatory process, correlated genes in this module may also be important.

Figure 3B overlays within‐module connectivity (point colour) and the volcano for dehydration versus control comparison including genes in the ‘lightcyan’ module. AVP had high intramodular connectivity in the ‘light cyan’ module with CAPRIN2 its closest neighbour in the network. We have previously identified this gene using an alternative network analysis tool as an important player in the rat SON^30^ and shown CAPRIN2 binds to the AVP mRNA and mediates an increase in the length of its polyA tail.^23^ Other genes with high connectivity include PCSK1 and PTPRN. These genes clearly respond strongly to the dehydration condition and may be central to how the jerboa SON orchestrates living in arid conditions.

### 3.5 Cross species comparison

These data are the first of their kind in the jerboa SON. How they fit into the context of mammalian osmoregulation is an important question that is addressed here by comparison with our previously published camel (*Camelus dromedarius* ^8^) and rat (*Rattus norvegicus* ^6^) datasets. These studies are summarised in Supporting Information.

Gene identifiers were consolidated to orthologues of a single species, namely rat, to aid cross‐species comparison. Figure S3 shows the data loss (duplication and failure to find a valid orthologue) when converting from jerboa and camel Ensembl gene identifiers to rat (Figure S3A) Ensembl gene identifiers.

The total number of genes with detectable expression across all samples (regardless of experimental group) in each species were as follows: jerboa (17,583), camel (15,230), and rat (15,576) (Figure S3). Note that lowly expressed genes in each dataset were excluded using a cut off of base mean <8 during DESeq2 processing. There are 9231 genes that are expressed in the SON of all three species and these were used as the working dataset for comparison of DEGs across species. A significant correlation was seen between ranked (by mean expression) gene sets between species (Figure S3).

**FIGURE 4:**
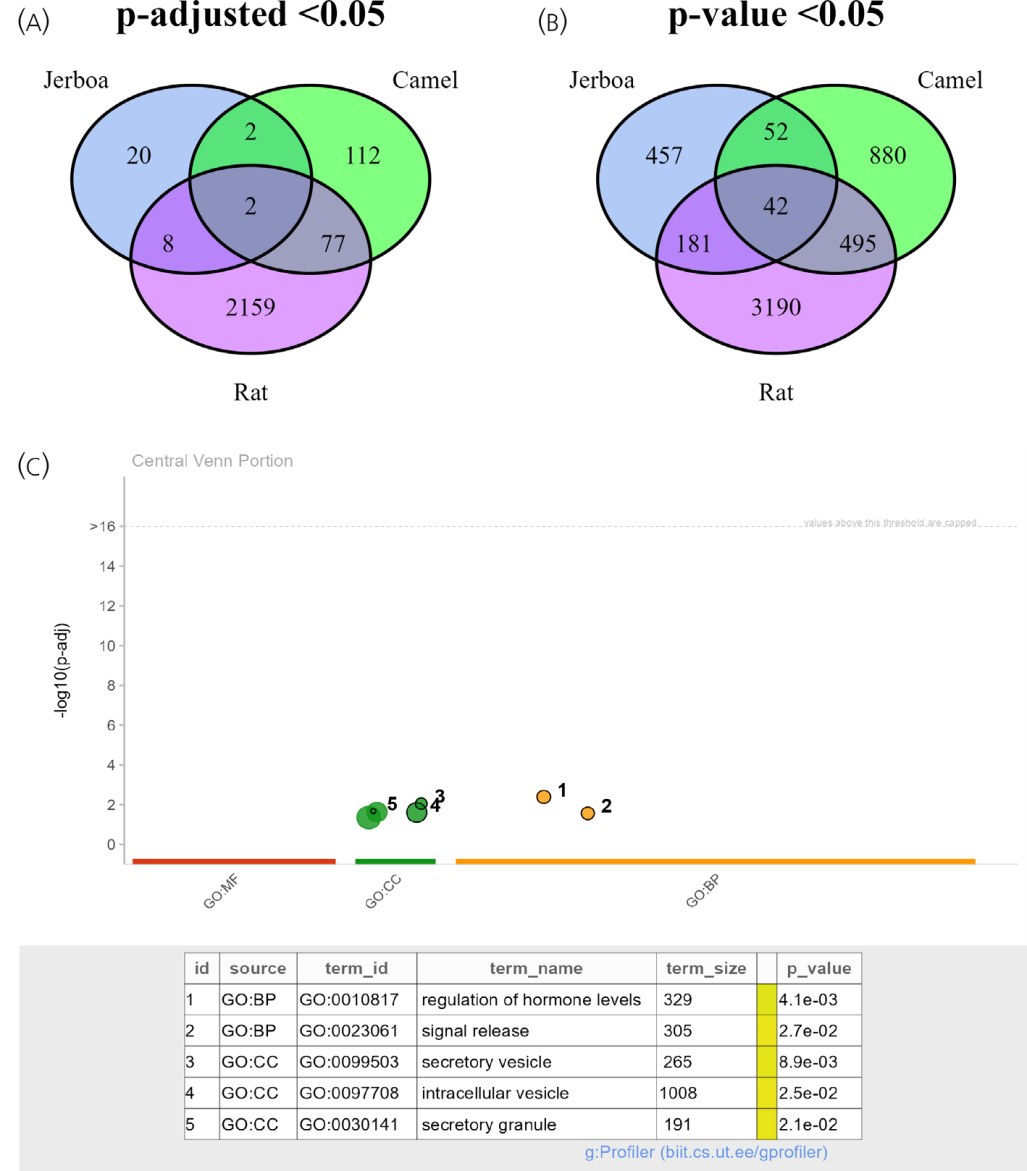
Comparison of DEGs in the SON of jerboa, camel, and rat after dehydration. Note the differing periods of water restriction and different technical biases for each species' analysis. (A) Cross species comparison of SON DEGs using a threshold of *p*_adj_ < 0.05. (B) Cross species comparison of SON DEGs using a threshold of *p*‐value <0.05. (C) Enriched GO terms from the DEG (*p* < 0.05) list shared across all three species. Highlighted terms relate to secretory processes.

For the control versus dehydration comparison, the jerboa data set contained 46 DEGs (*p*_adj_ < 0.05), of which 32 have recognised orthologues in rat. The camel data set contained 206 DEGs (*p*_adj_ < 0.05), of which 194 have recognised orthologues in rat. The rat dataset has 2246 DEGs (*p*_adj_ < 0.05). Figure 4A shows the overlap of DEGs in each species from the comparable gene list.

The rat had a higher proportion of DEGs (*p*_adj_ < 0.05, *p* < 0.05 shown in brackets) in the SON than the desert species. A total of 14.4% (25.1%) of genes were DEGs compared with 0.3% (6.4%) in the jerboa and 1.4% (10.6%) in the camel.

There were two genes that had significantly altered expression after dehydration in all three species with a threshold of *p*_adj_ < 0.05: PCSK1 and PTPRN.

The number of shared DEGs increases from 2 to 43 when using a p‐value threshold in place of a *p*_adj_ threshold (Figure 4B). Both these thresholds are arbitrary. But when using the *p*‐value threshold, the possibility of false positives is greater without multiple testing correction. However, this is mitigated somewhat if the gene is differentially expressed in three independent experiments across three different species.

When submitting the 43 shared DEGs (*p* < 0.05) from Figure 4B to ORA with a background list of the entire rat genome, multiple terms were enriched that referenced secretion pathways (Figure 4C) including regulation of hormone levels, signal release, secretory vesicle, intracellular vesicle, and secretory granule. The MCNs are secretory cells, so it stands to reason that secretory processes would be activated by dehydration.

## 4 DISCUSSION

This is the first dataset to explore the SON transcriptome of a desert rodent during water restriction. To give context, we have compared this new data to previously published data of a large xeric animal (camel) and a mesic model organism (rat).

We have shown that the jerboa has the canonical response to osmotic cues as AVP mRNA levels are increased in the SON by dehydration then recover to control levels when water is made available as has been shown in other desert rodents (*Meriones libycus*,^31^ *Meriones shawi*,^32^ *Taterillus gracilis*, and *Steatomys caurinus* ^33^).

Only 46 DEGs were found in the jerboa SON following 10/11 days water restriction. The rat SON had a proportionally greater number of DEGs despite the water restriction period being much shorter for rats (3 days) than jerboas (10/11 days) or camels (20 days). Furthermore, far fewer DEGs were found in the jerboa SON compared to the kidney.^13^ Interestingly, Blumstein and MacManes^34^ recently saw a notable lack of differential gene expression in the whole hypothalamus after water restriction in a desert rodent whilst other tissues, including the kidney, showed large changes in gene expression. In addition, the camel also follows this trend.^8^, ^35^

This may partly be a result of the differences in tissue composition. Blumstein and MacManes^34^ posit a lack of resolution in the diverse hypothalamus but our data in jerboa and previous data in the camel suggest the opposite. The SON sample is from a specific nucleus in the brain with relatively limited cell types compared to the diversity of the whole kidney organ. Single cell resolution of both tissues in desert animals would be the next logical step in understanding what is driving the difference in magnitude of DEGs.

This suggests that the hypothalamus, and more specifically the SON, has mechanisms of water balance control in xeric species that are orchestrated by the transcription of a focussed group of influential genes and that this group is more concentrated than in mesic species. It follows that shared DEGs across species are among the most important in the SON for this response.

Two genes were significantly (*p*_adj_ < 0.05) altered in the SON of the jerboa, rat, and camel following osmotic challenge: PCSK1 and PTPRN.

PCSK1 (ENSRNOG00000011107) was also a DEG in microarray data in the mouse^7^ and rat^4^ SON. PCSK1 encodes the protein PC1/3 (previously known as PC1, PC3, or SPC3) and is expressed in secretory cells such as those in the rat SON.^36^ PCSK1 is involved in the prohormone processing of a variety of endocrine peptide products including vasopressin, oxytocin,^37^ ACTH,^38^ GLP‐1,^39^ POMC,^40^ and insulin.^41^, ^42^ See supplemental table 1 in Stijnen et al.^43^ for a list of possible substrates. Disruption of PCSK1 in humans manifests in a variety of endocrine disorders.^43^

An example of PCSK1 and its prohormone cleaving function is in the beta (and alpha) cells of the pancreas where PCSK1 processes prohormone peptides into GLP‐1 and insulin.^44^ Interestingly, PCSK1 expression in alpha cells can be initiated by liraglutide, a GLP1R agonist. GLP1R is a gene that is regulated in Jerboa, camel, and rat SON during dehydration. Consistent regulation across species during dehydration and evidence of processing a variety of prohormones suggests PCSK1 is an important component of the general secretory process.

PTPRN (IA‐2/ENSJJAG00000018101) is a protein tyrosine phosphatase receptor involved in signalling at the plasma membrane and vesicle exocytosis. PTPRN is expressed in endocrine secretory cells, both in the brain and pancreas. Specifically, PTPRN has been shown to be involved in the membrane of secretory granules.^45^ In an insulin secreting mouse cell line, overexpression of PTPRN increased secretory vesicle number and the insulin content of cells, whereas knockdown markedly reduced insulin secretion.^46^ IA2 antibodies (often used to test for type 1 diabetes autoimmunity) were found in 70% of human patients with central diabetes insipidus.^47^ These previous insights alongside the consistent regulation during dehydration across species make PTPRN an interesting candidate as a pivotal part of the secretory system in endocrine cells.

## 5 LIMITATIONS

An example of the pitfalls of investigating non‐model organisms is the absence of AVP and CAPRIN2 in the camel dataset due to missing annotation (further investigation provided evidence of expression^8^). This suggests that the gene list produced by the cross‐species comparison is not exhaustive and more targets may appear as annotations improve. Another source of potential false negative DEGs is the acclimatisation period in the experimental design. In order to control for individual differences in animals prior to the physiological challenge this was required, but could have led to gene expression changes that do not reflect natural conditions as access to water was freely available during the acclimatisation period.

The transcriptomes of samples in Jerboa and camel in SON and kidney generated from the rehydration condition appear to be more variable than the control or dehydration conditions. A possible source of this variation is the sequence of recovery in the rehydration condition. The jerboa appears to have successfully recovered from water restriction as shown by the similarity in gene expression between euhydrated and rehydrated conditions. The speed of these changes is unknown as the rehydration condition is an understudied addition to experiments exploring water balance and should be considered in future.

## 6 CONCLUSION

In the SON of xeric species, fewer transcriptional changes are seen after osmotic challenge when compared to other tissues and mesic species. PCSK1 and PTPRN are conserved osmotically responsive genes in the SON (and kidney) of jerboa, camel, and rat, suggesting they are crucial components in the systemic response to dehydration.

## AUTHOR CONTRIBUTIONS

**Abdulaziz N. Alagaili:** Conceptualization; methodology; writing – review and editing; supervision; investigation; resources; project administration; funding acquisition. **Michael P. Greenwood:** Conceptualization; methodology; writing – review and editing; supervision; project administration; funding acquisition. **Nabil Amor:** Investigation; validation; writing – review and editing. **Benjamin T. Gillard:** Conceptualization; methodology; data curation; formal analysis; validation; investigation; visualization; writing – original draft; writing – review and editing; project administration; software. **David Murphy:** Writing – review and editing; conceptualization; supervision; methodology; resources; project administration; funding acquisition.

## FUNDING INFORMATION

The funder had no role in the design, data collection, data analysis, and reporting of this study. This research was generously supported by grants from the Leverhulme Trust (RPG‐2017287) to Benjamin T. Gillard, David Murphy, and Michael P. Greenwood; the BBSRC (BB/R016879/1) to David Murphy and Michael P. Greenwood; the EPSRC (EP/K008250/1) to David Murphy; and also funded by Ongoing Research Funding Program (ORF‐2025‐602), King Saud University, Riyadh, Saudi Arabia to Abdulaziz N. Alagaili. The Biotechnology and Biological Sciences Research Council‐SWBio DTP program (BBSRC BB/M009122/1) supported Benjamin T. Gillard.

## CONFLICT OF INTEREST STATEMENT

The authors declare no conflicts of interest.

## Supporting information


**Table S1.** Summary of methods used for each species dataset used in this analysis.
**Figure S1.** Validation of Jerboa SON RNAseq by qPCR. Correlation between RNAseq and qPCR results in dehydration vs. control (A) and rehydration vs. control (B) comparisons. Spearman correlation used. B‐H Relative expression of selected genes in the Jerboa SON assayed by qPCR. Significance calculated by Tukey post hoc test. NS = Not significant, * = <0.05, ** = <0.01, *** = <0.001.
**Figure S2.** AVP concentration (picograms per gland) in the Jerboa PP in each experimental condition (Control, Dehydration, Rehydration). Measured by ELISA. Significance between each condition was calculated by one‐way ANOVA with Tukey post hoc test. Sex had no significant influence on PP AVP content (*p* = 0.72, F = 0.13, df = 1) so an ANOVA was performed using condition as the independent variable. Concentration per gland was significantly changed by condition (ANOVA, p = <0.001, F = 10.98, df = 2). A Tukey post hoc test found a significant reduction in AVP concentration per gland after water restriction (p = <0.001) which was recovered after rehydration (*p* = 0.79) compared to control. NS = Not significant, * = <0.05, ** = <0.01, *** = <0.001.
**Figure S3.** Conversion of gene identifiers found in the SON between species. Finding equivalent gene identifiers in a different species using the gorth function creates duplicate identifiers and can fail to find an orthologues in the target species at all (NAs). A Loss of data through duplicates and missing orthologues when converting to human Ensembl identifiers from jerboa, camel, and rat. B Loss of data through duplicates and missing orthologues when converting to rat Ensembl identifiers from jerboa and camel. C Detectable gene expression in the SON of each species and the overlap of genes. Brackets denote the number of detectable genes in the SON for each species before identifier conversion. D‐F Comparison of SON expression between species. Genes are ranked by mean expression. All three species had correlated expression by rank. Dups = duplicates.

## Data Availability

The data that support the findings of this study are openly available at https://www.ncbi.nlm.nih.gov/geo/ (reference number GSE242975). The data from all tissues and species that have been sequenced by our lab can be accessed at https://app-shiny.services.bris.ac.uk/jerboaKidneyGeneSearch/.
