# Supplementary material for: Transcriptomic changes in the jerboa supraoptic nucleus during osmotic stress: PCSK1 and PTPRN changes are conserved across desert and mesic species
Source: J Neuroendocrinol. 2026 Jul 26;38(8):e70233. doi: 10.1111/jne.70233 (PMC13402333; doi:10.1111/jne.70233)
Supplement: Supplementary file 1 — Table S1. Summary of methods used for each species dataset used in this analysis. Figure S1. Validation of Jerboa SON RNAseq by qPCR. Correlation between RNAseq and qPCR results in dehydration vs. control (A) and rehydration vs. control (B) comparisons. Spearman correlation used. B‐H Relative expression of selected genes in the Jerboa SON assayed by qPCR. Significance calculated by Tukey post hoc test. NS = Not significant, * = <0.05, ** = <0.01, *** = <0.001. Figure S2. AVP concentration (picograms per gland) in the Jerboa PP in each experimental condition (Control, Dehydration, Rehydration). Measured by ELISA. Significance between each condition was calculated by one‐way ANOVA with Tukey post hoc test. Sex had no significant influence on PP AVP content (p = 0.72, F = 0.13, df = 1) so an ANOVA was performed using condition as the independent variable. Concentration per gland was significantly changed by condition (ANOVA, p = <0.001, F = 10.98, df = 2). A Tukey post hoc test found a significant reduction in AVP concentration per gland after water restriction (p = <0.001) which was recovered after rehydration (p = 0.79) compared to control. NS = Not significant, * = <0.05, ** = <0.01, *** = <0.001. Figure S3. Conversion of gene identifiers found in the SON between species. Finding equivalent gene identifiers in a different species using the gorth function creates duplicate identifiers and can fail to find an orthologues in the target species at all (NAs). A Loss of data through duplicates and missing orthologues when converting to human Ensembl identifiers from jerboa, camel, and rat. B Loss of data through duplicates and missing orthologues when converting to rat Ensembl identifiers from jerboa and camel. C Detectable gene expression in the SON of each species and the overlap of genes. Brackets denote the number of detectable genes in the SON for each species before identifier conversion. D‐F Comparison of SON expression between species. Genes are ranked by mean exp [file JNE-38-e70233-s001.zip › jne70233-sup-0001-Supinfo.docx]

Supplemental Materials

DESeq2 output DESeq2 output for comparisons between control, dehydration, and rehydration jerboa SON transcriptomes and VST normalised expression matrix. Human readable gene names have been added based on the Ensembl IDs using the gProfiler2 R package. Files JacJacSON_DESeq2VSTNormalisedExpressionDataConplusSex.csv, JacSON_DehydrationvsControlConplusSex.csv, JacSON_DehydrationvsRehydrationConplusSex.csv,

JacSON_RehydrationvsControlConplusSex.csv, JacSON_SexDifferencesAdditive.csv

WGCNA module gene lists Genelists for each WGCNA module generated from the jerboa SON transcriptome. xlsx file WGCNAModuleGeneLists_SON.xlsx.

Summary of published studies used for cross species comparison. In Pauža et al., 2021, the SON (bilateral) of the rat was dissected and sequenced after 3 days water restriction and compared to euhydrated samples. Animals were killed by striking of the cranium then decapitation. This is considered the optimal method to limit confounding effects on gene expression in the SON. Library preparation included poly-A selection and sequencing was performed by Illumina NextSeq for >35 million paired end reads per sample. STAR was used for read alignment to the genome. Expression after osmotic challenge was compared across two strains of rat with 22.43% of Wistar (RNAseq derived) and 40.52% of Sprague-Dawley (microarray derived) DEGs overlapping with the other strain and a strong correlation (Spearman 0.78, r^2^ = 0.61) in the log2 fold change of these genes. RNAseq was found to be more sensitive than microarray (i.e. more DEGs were found when expression differences were smaller between groups). The publication of many previously unstudied genes was intended to encourage the exploration of genes with no known function in the SON.

In Lin et al., 2022, the transcriptome of the camel SON after dehydration was investigated. This paper included xeric species in a comparison of camel SON transcriptomic response to that of the rat. 80 overlapping dehydration DEGs were found between the species accounting for 3.56% and 62.5% of total rat and camel DEGs respectively. As with the comparison of rat strains in Pauža et al., 2021, there was a strong correlation in log 2 fold change between DEGs in the camel and rat SON (Spearman 0.716, p<0.0001). Conserved DEGs between the species were enriched for the GO term “Response to cAMP” suggesting a conserved pathway in the SON response to dehydration.

| Species Tissue Sex Age | Dehydration period (Days) Kill method Extraction time Library preparation RIN |
| --- | --- |
| jerboa SON Male and Female NA | 10/11 Ether overdose 10am-12pm Poly-A 9.3 |
| Rat SON Male 13 weeks | 3 Cranium strike and decapitation 10am-12pm Poly-A 8.5 |
| Camel SON Male 4-5 years | 20 Abattoir, Halal 9am-5pm Ribo depletion 6.3 |

Supplementary Table 1: Summary of methods used for each species dataset used in this analysis

| Species | Tissue | Sequencing | Read depth | Analysis Pipeline | GSE accession |
| --- | --- | --- | --- | --- | --- |
| jerboa | SON | Illumina NextSeq500 | 24 million | BBDuk, Rsubread, FeatureCounts, DESeq2 | GSE242975 |
| Rat | SON | Illumina NextSeq | 35 million | STAR, FeatureCounts, DESeq2 | GSE175461 |
| Camel | SON | Illumina NextSeq500 | 22.2 million | BBDuk, STAR, FeatureCounts, DESeq2 | GSE198577 |


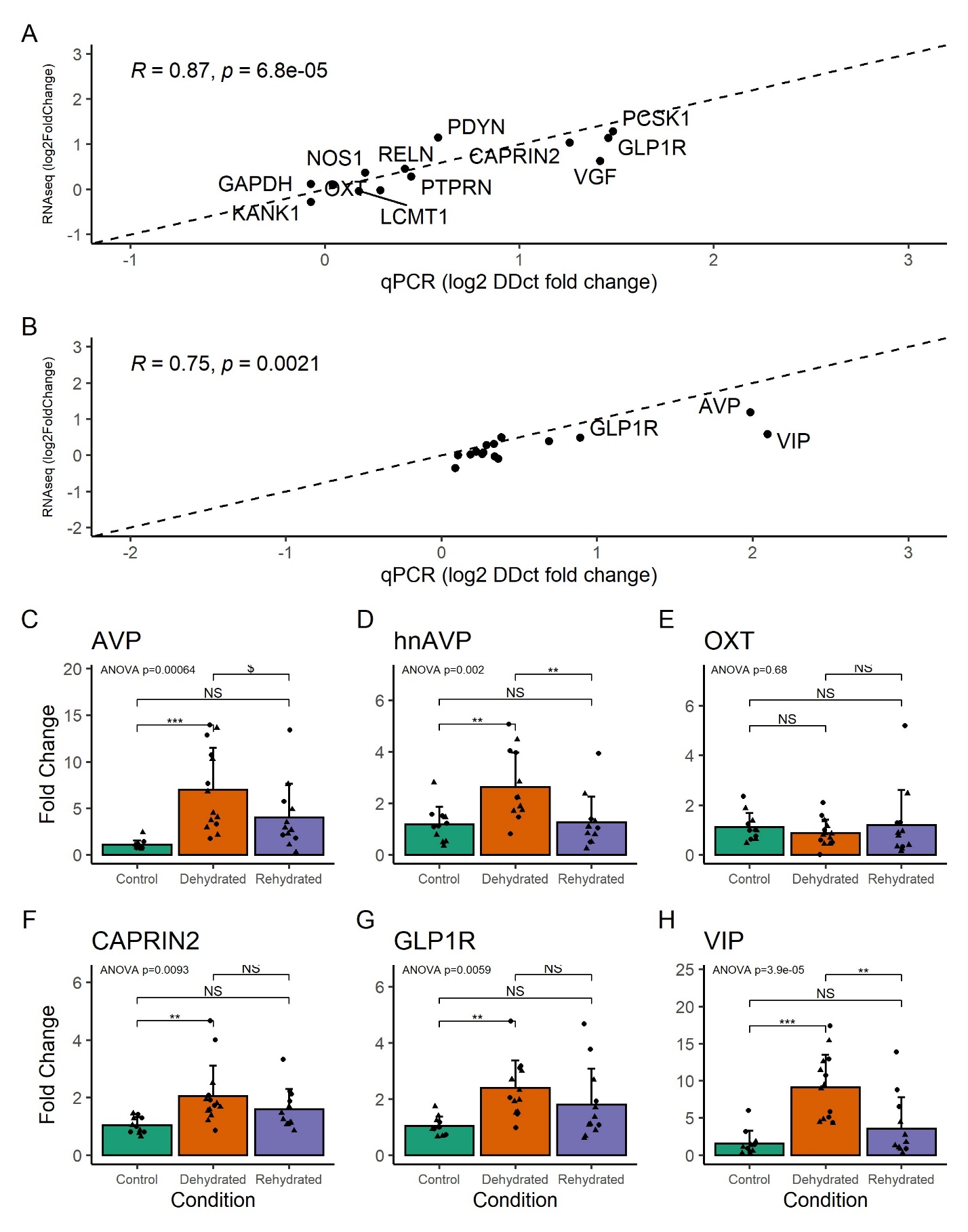


Supplementary Figure 1: Validation of Jerboa SON RNAseq by qPCR. Correlation between RNAseq and qPCR results in dehydration vs control (A) and rehydration vs control (B) comparisons. Spearman correlation used. B-H Relative expression of selected genes in the Jerboa SON assayed by qPCR. Significance calculated by Tukey post hoc test. NS = Not significant, * = <0.05, ** = <0.01, *** = <0.001


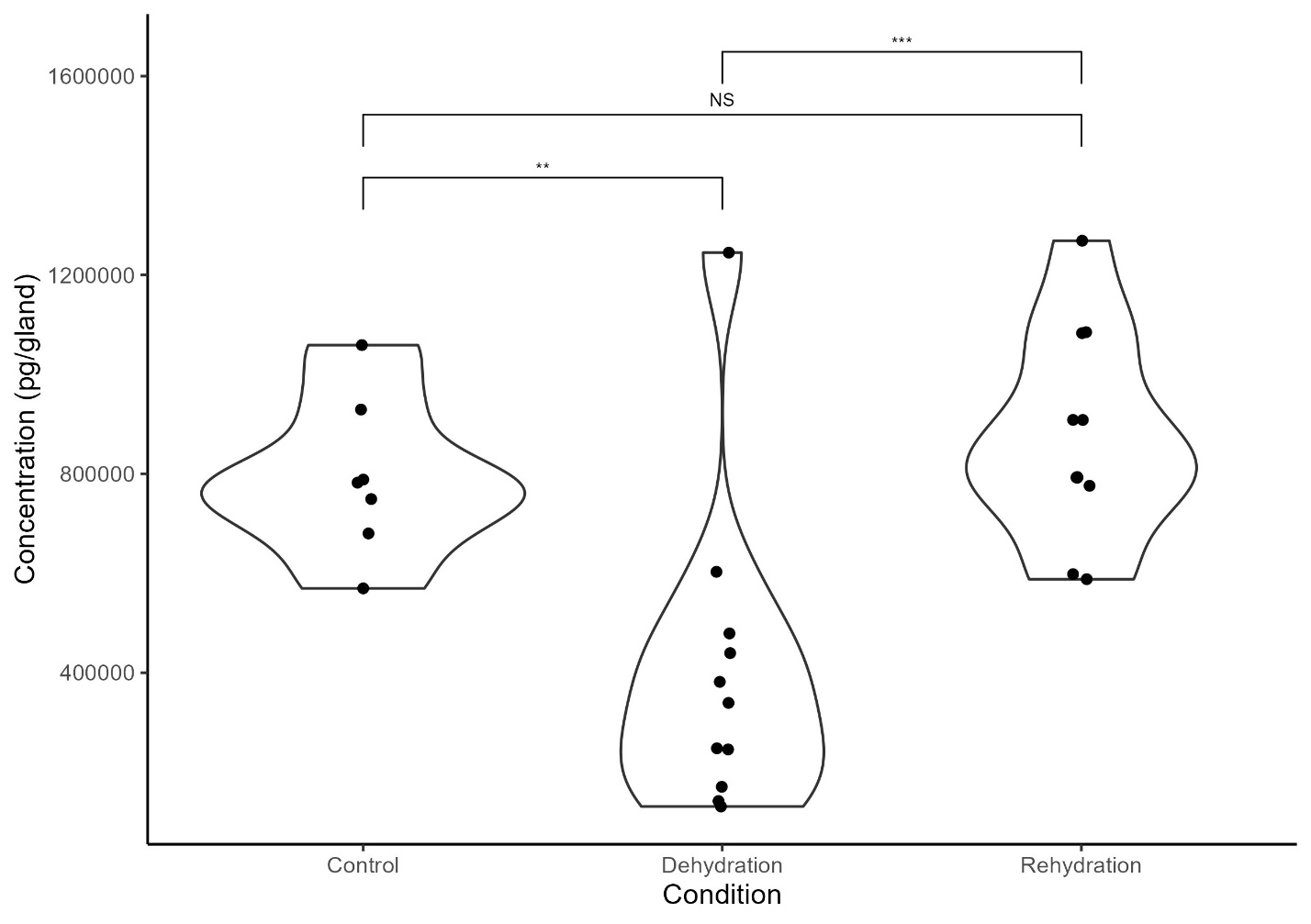


Supplementary Figure 2: AVP concentration (picograms per gland) in the Jerboa PP in each experimental condition (Control, Dehydration, Rehydration). Measured by ELISA. Significance between each condition was calculated by one-way ANOVA with Tukey post hoc test. Sex had no significant influence on PP AVP content (p=0.72, F=0.13, df=1) so an ANOVA was performed using condition as the independent variable. Concentration per gland was significantly changed by condition (ANOVA, p=<0.001, F=10.98, df=2). A Tukey post hoc test found a significant reduction in AVP concentration per gland after water restriction (p=<0.001) which was recovered after rehydration (p=0.79) compared to control. NS = Not significant, * = <0.05, ** = <0.01, *** = <0.001


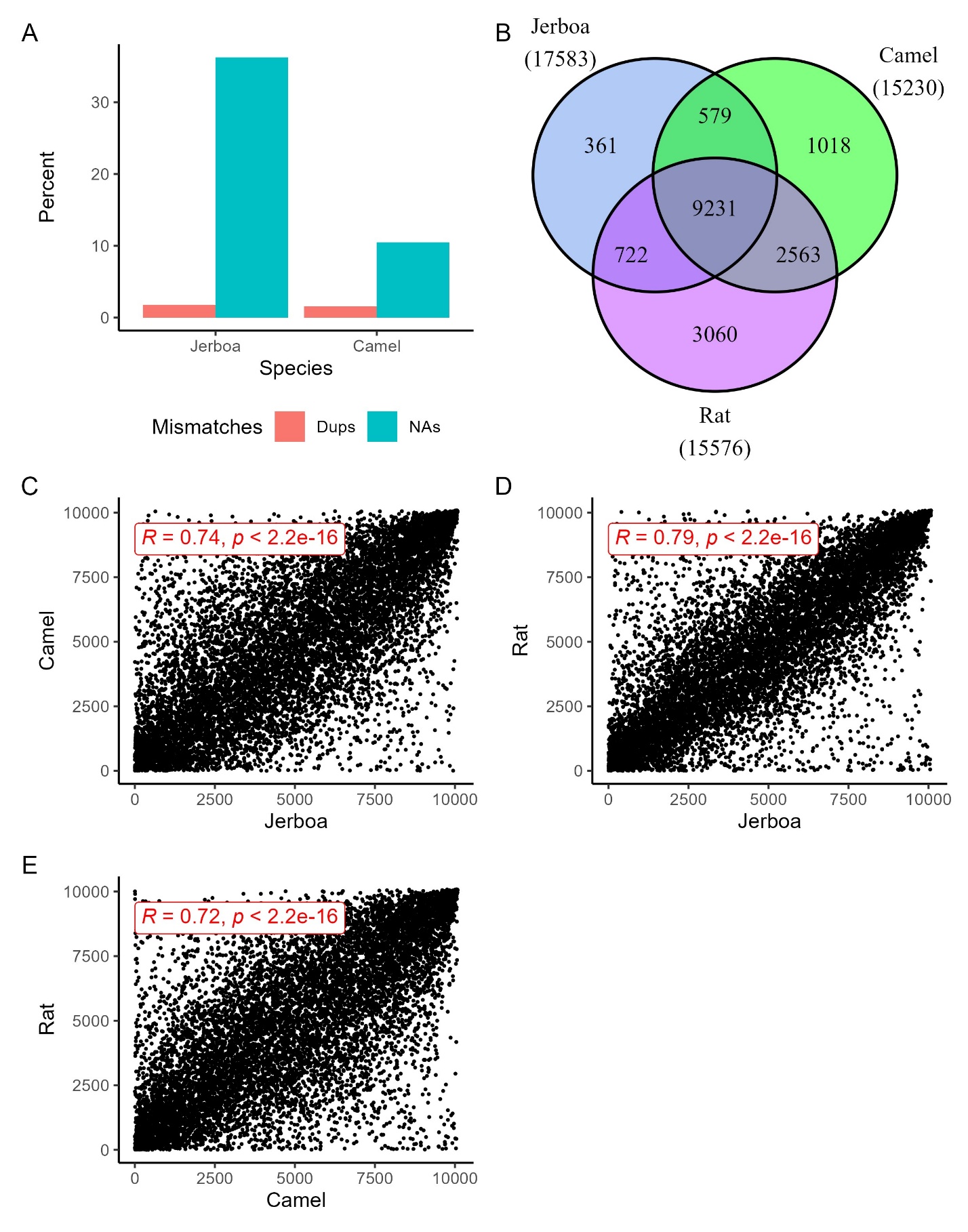


Supplementary Figure 3: Conversion of gene identifiers found in the SON between species. Finding equivalent gene identifiers in a different species using the gorth function creates duplicate identifiers and can fail to find an orthologues in the target species at all (NAs). A Loss of data through duplicates and missing orthologues when converting to human Ensembl identifiers from jerboa, camel, and rat. B Loss of data through duplicates and missing orthologues when converting to rat Ensembl identifiers from jerboa and camel. C Detectable gene expression in the SON of each species and the overlap of genes. Brackets denote the number of detectable genes in the SON for each species before identifier conversion.23 D-F Comparison of SON expression between species. Genes are ranked by mean expression. All three species had correlated expression by rank. Dups = duplicates.
